# Representation of Women and Women's Health in Australian Medical School Course Outlines, Curriculum Requirements, and Selected Core Clinical Textbooks

**DOI:** 10.1089/whr.2023.0037

**Published:** 2024-04-05

**Authors:** Lea Merone, Komla Tsey, Darren Russell, Cate Nagle

**Affiliations:** ^1^ James Cook University, College of Healthcare Sciences, Townsville, Queensland, Australia.; ^2^ Cairns Voluntary Assisted Dying Service, Cairns and Hinterland Hospital and Health Service, Cairns North, Queensland, Australia.; ^3^ Cairns Sexual Health Service, Cairns North, Queensland, Australia.

**Keywords:** gender, sex, clinical textbooks, curriculum, medical education

## Abstract

**Background:**

Historically, medical research has, outside of reproductive health, neglected the health needs of women. Medical studies have previously excluded female participants, meaning research data have been collected from males and generalized to females. Knowledge gained from research is translated to clinical education and patient care, and female exclusion may result in gaps in the medical school curricula and textbooks.

**Materials and Methods:**

This study involved a desktop review of the *Australian Medical Council Standards for assessment and accreditation of primary medical programs,* the online publicly available Australian medical school course outlines, and finally, an analysis of the recommended textbooks.

**Results:**

There is no fixed or explicit requirement to include women's health in Australian medical school curricula. Medical school course outlines do not adequately include women's health; similarly, clinical medicine textbooks do not account for sex and gender differences.

**Conclusion:**

Important sex and gender differences in medicine are not reflected adequately in the medical school course outlines, curricula, or clinical textbooks. This may have significant consequences on women's health.

## Background

Historically, medical research has, outside of reproductive health, neglected the health needs of women.^1^ Medicine, acknowledged as both science and art, is as much social and cultural as it is scientific.^2^ Gender is a social construct^3^ and as medicine developed throughout history, it has in many ways enforced the constructed gender divisions, from Aristotle, who defined women as anatomically “mutilated” men,^4^ to Freud and Breuer, who utilized female case studies to develop the psychoanalytic concept of hysteria.^5^

Even in the relatively modern era, myths surrounding the female body and health have persisted.^2^ Such sex and gender myths today may manifest as a sex and gender gap between female and male health care; women wait longer than men for diagnosis^6^ and pain relief. Furthermore, women are more likely than men to be misdiagnosed or discharged during serious medical events.^6–8^ Previously, medical studies have excluded female participants, meaning research data have been collected from males and generalized to females^9^ and intersex people.^10^

Medical studies are underpinned by assumptions about sex and gender that are in keeping with social norms.^11^ Research has demonstrated that biological and socioeconomic differences between women and men contribute to the variation in health and disease between women and men.^12^ Medicine as an institution has been accused of being “gender blind” and considering the sex and/or gender of the patient to be largely irrelevant to clinical care. Medicine largely assumes women and men to be identical in terms of disease presentation, disease course, investigations, and response to treatment. In addition, the ideology, biases,^13^ and preconceptions of clinicians regarding gender roles and gender expression may negatively influence treatment and outcomes.^14^ Combined, this simultaneous gender blindness and social preconceptions of women can have adverse impacts on the experiences of women patients.^15,16^

There are many advocates for incorporating gender medicine, defined as “a study of differences in men's and women's normal function and in their experiences of the same disease,” into medical education.^17^ Despite efforts to implement gender medicine into medical school curricula across Europe and the United States, overall appetite for this has been poor.^17^ Yet, despite this, there is increasing recognition that sex as a biological variable needs to be considered when educating clinicians and future clinicians on evidence-based diagnoses and treatments.^18^

Knowledge gained from research is translated to clinical education and patient care^19^; therefore gaps in clinical research will inevitably be translated into gaps in the medical school curricula and textbooks. This may adversely impact women's health. In addition, lack of inclusion of women and people of color enhances implicit biases among clinicians, positioning the white 70 kg male as “normal” and implying anything other is “abnormal.”^20^ Given the importance of the research evidence base to both clinical practice and education, it is imperative that information is up to date and accurate, and accounts for differences in sex and gender.^21^ Sex and gender gaps may be prominent in medical school course outlines and curricula, impacting upon students' implicit gender views^22^ and potentially affecting patient care, particularly that of women.

Gaps in knowledge of sex and gender difference in disease may also be observable in medical textbooks. A clinical medicine textbook is one that is focused on the diagnosis, investigation, and management of disease. Use of clinical medicine textbooks is common in medical schools across Australia. Clinical guidelines and textbooks are guided by the literature, and there is a known lag of 17 years between publication and translation to patient management through clinical guidelines.^23^ Although efforts are being made to reduce this gap,^24^ it is important to note as even as women participation in clinical trials increases, there will be a significant delay before the results of these are reflected in the medical school curricula, textbooks, and patient care.

There are currently 20 medical schools across Australia, each with a course outline and learning outcomes available online. The aim of this two-phase quantitative study is to explore the representation of women and women's health in medical school course outlines, the *Standards for Assessment and Accreditation of Primary Medical Programs by the Australian Medical Council* (2012),^25^ and recommended clinical medicine textbooks, with attention to gender and sex differences in the social circumstance, presentation, diagnosis, and medical care of women patients.

## Materials and Methods

This study involved a desktop review of the *Australian Medical Council Standards for assessment and accreditation of primary medical programs*, online publicly available medical school course outlines, and finally, a textbook content analysis of the recommended textbooks.

### Phase 1: Review of the *Australian Medical Council Standards for assessment and accreditation of primary medical programs* and online course outlines

#### Data collection

The *Standards for Assessment and Accreditation of Primary Medical Programs by the Australian Medical Council* (2012),^25^ the criteria against which medical education programs are evaluated, were reviewed, and data were collected on:

- Whether a women's health unit is stipulated in the guidelines as a requirement for all medical courses
- If requirements of any women's health components include nonreproductive elements
- If requirements of any women's health components include acknowledgment of the differing socioeconomic circumstances of women and men.

A review was also undertaken, collating publicly available course outline information from Australian universities providing entry to practice programs for medicine. The data collection tool was developed using the Queensland Government Gender Analysis toolkit.^26^ This data collection tool gathered information on the presence or absence of inclusion of women in course outlines and whether that inclusion extended beyond sexual or reproductive health for women.

Data were collected on the number and proportion of medical schools that

- Provide an online course outline
- Designate women's health courses or learning outcomes that are centered on the reproductive system
- Designate women's health courses or learning outcomes that are not centered on the reproductive system
- Designate women's health courses or learning outcomes that highlight medical differences between women and men
- Designate women's health courses or learning outcomes that highlight social and cultural differences between women and men.

#### Data analysis

Descriptive statistical analysis of these data summarized the extent to which women's health is included in the *Standards for Assessment and Accreditation of Primary Medical Programs by the Australian Medical Council* and the Australian Medical Schools' course outlines. This included requirements for women's health components within medical school curricula, number and proportion of medical schools that had a designated women's health course, and number and proportion that included women's health outside of sexual or reproductive health (such as obstetrics and gynecology rotations).

### Phase 2: Textbook Analysis

Analysis of medical school-recommended textbooks was guided by a data collection form modified from that developed by Alexanderson et al.^2^7 The table of contents and indices of each book was hand searched for “gender,” “sex,” “male,” “female,” “men,” and “women” to determine if there is specific inclusion of women's health outside of reproductive medicine.

Informed by results of a cross-sectional analysis,^28^ the specialties with the least representation of women in research were selected for analysis: cardiology, gastroenterology, and nephrology. Two diseases were selected from each specialty: coronary heart disease (CHD) and heart failure; irritable bowel syndrome (IBS) and peptic ulcer disease; and chronic kidney disease (CKD) and renal calculi. The content for each of these six conditions was examined in each of the six textbooks (for a list of textbooks, please see Table 1).

**Table 1.** Clinical Medicine Textbooks Recommended by 10 Australian Medical Schools and the Frequency of Which the Textbook was Recommended

Textbook title and edition (year of publication)	Frequency of recommendation
**Kumar and Clarke Clinical Medicine, 8th edition (2012)**	5
**Harrison's Principles of Internal Medicine, 18th edition (2011)**	4
**Oxford Handbook of Clinical Medicine, 9th edition (2014)**	4
**Davidson's Principle and Practice of Medicine, 22nd edition (2014)**	2
**Stewart Core Clinical Medicine (2010)**	1
**Gibson's Essential Clinical Medicine (2009)**	1
**Toronto Notes Essential Medicine Notes (2020)**	1

These conditions were selected as per similar work by Dijkstra et al.,^29^ as they are,

1. Common within the selected specialty, for example, coronary or atherosclerotic heart disease for cardiology.
2. Represent a major public health issue, for example, have significant population prevalence across all sexes and genders.
3. Involve potentially relevant biomedical, psychosocial, and cultural sex- and gender-related issues.

#### Data collection

Alexanderson et al. developed a framework specifically for gender analysis in medical textbooks.^21^ A version of this framework was adapted for this study, eliminating sections that were irrelevant, for example, sections that pertained to public health medicine and questions about references to author and scientist gender. In addition, in part, the methods of Parker et al. were employed to examine gender representation in images used in medical textbooks.^30^ Parker et al. examined the ratio of females: males represented in images and case studies, and where relevant, examined the narrative of the image to determine if females are more frequently shown to be in domestic-type roles and males more frequently shown to be in professional roles.^22^ For each textbook, data were collected on the following:

- Number of words in the contents and index referring to female sex and/or gender, male sex and/or gender, and sex and/or gender more generally
- Presentation of (in terms of sex and/or gender):
  o disease epidemiology
  o disease etiology
  o risk factors for the disease
  o treatment for the disease and response to treatment
  o specific mention of women's symptoms
- Representation of sex and/or gender in textbook images
  o Number of images depicting anatomical females and males
  o Images positioning people in stereotyped roles (*e.g.*, women in domestic roles and men in professional roles)

#### Textbook content analysis

Descriptive statistics were conducted to determine if there is difference between representation of women and men both within and between recommend textbooks. This included information on whether gender differences in disease etiology, risk factors, presentation, investigation, and management were included and additionally, on whether consideration was given to the different socioeconomic circumstances of women and the impacts this may have on their health.

Of note, textbooks were inconsistent in the information provided for each disease, for example, one textbook may provide epidemiology for CHD, but not for IBS. Thus, for the purposes of this study, conditions are considered sections of books, of which there are 36 in total (six conditions across six textbooks).

#### Textbook image analysis

The data were used to determine the relative frequencies of men and women represented in images and whether gendered representations were stereotyped. This included examination of representation of female bodies and presence of stereotyped images (men in professional roles and women in domestic roles).

## Results

### Phase 1: Review of the *Australian Medical Council standards for assessment and accreditation of primary medical programs* and online course outlines

The *Australian Medical Council standards for assessment and accreditation of primary medical* programs^25^ was reviewed utilizing a modified version of the curriculum analysis data collection tool. There is no explicit mention or direction regarding a requirement for specific course content on women's health in the assessment guide or procedure provided. Women's health was listed as a potential clinical placement; however, no requirement for this is clear in the document. There was no recommended course content for women's health, nor acknowledgment of the different social circumstances specifically for women.

Of the 20 Australian medical schools, there was 1 medical school that provided insufficient online course outline information for analysis and was thus excluded. Of the 19 medical schools that were included, 84% (*n* = 16) stated they had a course pertaining to women's health. Of these 16, 14 of the courses were listed as clinical rotations in women's health or obstetrics and gynecology between years 3 and 5 of medical school.

There was one course that stated there was a women's health workshop; however, no further detail was provided, and one course that provided two elective subjects in women's health in years 1 and 5, again with no further detail provided. Of the 16 courses, only 2 medical schools provided sufficient information regarding learning outcomes for full assessment of the inclusion of women's health. None of the course information or outline provided appeared to focus on women's health or social circumstances outside of reproductive medicine or obstetrics and gynecology.

### Phase 2: Textbook analysis

Of the 20 medical schools in Australia, 10 provided their recommended clinical textbook list online. Table 1 outlines the list of books provided and the frequency of recommendation.

Six textbooks out of the recommended eight were included in the analysis, one textbook was unavailable to borrow or purchase and one textbook (Stewart Core Clinical Medicine, 2010) was deemed unsuitable for the analysis owing to its unusual structure and format; diseases were not discussed as separate entities and it was not possible to conduct the analysis. One textbook did not include a chapter or section on renal calculi; this was thus categorized as “not presented” across all categories.

#### Textbook content analysis

No clinical textbook included in this study had a dedicated chapter or section on women's health. The median number of words referring to women, men, and gender in the index across all the textbooks included is presented in Table 2.

**Table 2.** Median Number of Words Referring to Women, Men, and Gender Across the Indexes of All Included Textbooks

Words referring to sex and/or gender	Median number of words (IQR)
**Referring to women/female sex and gender**	2 (22.5)
**Referring to men/male sex and gender**	1 (13)
**Referring to gender**	1 (5)

IQR, interquartile range.

#### Presentation of disease in clinical textbooks

A total of 36 sections—1 for each of the 6 diseases, were examined within 6 textbooks. All six textbooks implied that women and men have the same symptoms for all the analyzed diseases, without acknowledgment of how women and men might present differently (although one textbook did state, in the case of CHD, that women are more likely to have atypical chest pain), recommended the same treatments, and predicted the same consequences and natural history of disease for women and men. No textbook acknowledged the differing social circumstances for women and men as contributing factors to disease and response to treatment, and no textbook stated whether their recommendations were based on research from women and men or men alone.

Across the six diseases, most textbooks presented disease etiology, risk factors, and symptoms as the same for women and men and did not consider disease pathology or presentation to differ between the sexes (Table 3).

**Table 3.** Descriptive Statistics Representing the Presentation of Sex Data Across All the Six Specified Sections of the Examined Medical Textbooks

Area	Sex data presented	Number of sections (out of a total of 36 across 6 textbooks)
**Epidemiology/prevalence**	Sex (females and males separately)	8
	Total prevalence (males AND females combined)	17
	Female prevalence only	1
	Male prevalence only	1
	Not presented	10
**Etiology**	Sex (females and males separately)	0
	Presented females and males as the same	35
	Not presented	1
**Disease risk factors**	Sex (females and males separately)	0
	Presented females and males as the same	19
	Not presented	17
**Response to treatment**	Sex (females and males separately)	1
	Presented females and males as the same	35
	Not presented	1
**Specific mention of women's symptoms**	Yes	1
	No	35

##### Epidemiology

Epidemiology was sometimes presented by sex (*n* = 8 out of 36 sections). One textbook, when describing IBS, provided the total epidemiology (males and females combined), followed by the epidemiology for females only. One textbook listed IBS as a functional disease.

##### Etiology and clinical features

Disease etiology was consistently presented across all textbooks as one entity (*n* = 35 out of 36 sections, and 1 section that did not present this information), with no difference specified between females and males. One textbook noted that females and males have different patterns of CHD; females are more likely to have small-vessel disease, compared with males who are more likely to have large-vessel disease. Another textbook noted differences in 24-hour urinary calcium excretion between females and males in the etiology of renal calculi.

##### Disease risk factors

Risk factors for disease were not presented in nearly 50% of cases (*n* = 17 out of 36). When presented, risk factors were not differentiated by sex or gender (*n* = 19 out of 36). Four textbooks presented male sex as a risk factor for certain CHD and one textbook listed male sex as a risk factor for heart failure, but one acknowledged this higher risk in men was comparative only to premenopausal women. Another textbook listed male sex as an “alarm” symptom in IBS and suggested searching for an organic cause of disease.

##### Treatment and response to treatment

Treatment and response to treatment were most frequently provided as universal across females and males (*n* = 35 out of 36 sections); however, one textbook stated that at angiography for females with diagnosed CHD, the coronary arteries are more likely to appear normal without clear mechanism or understanding of why this may be, rendering this treatment unhelpful.

#### Textbook image analysis

Across the 6 specialties in 6 textbooks, there were 10 relevant chapters with no image at all and 22 that contained images without people (*e.g.*, graphs, flow charts, radiology images, electrocardiogram [ECGs]). There was one photo of a patient undergoing a scan, their sex was not able to be determined. One image demonstrated an illustration of a male body as the norm and another image was a gender-neutral illustration. There was no image portraying gender stereotyping.

## Discussion

Within the online content reviewed, the lack of emphasis on women's health is clear; most medical schools provide a clinical rotation in obstetrics and gynecology, with no other mention of women's health or social situations, particularly outside of reproductive medicine. This has been observed historically throughout medicine, with women's health problems being attributed to their different genitalia and reproductive organs.^2^ This is in keeping with other curricula analyses conducted in the United States^31,32^ and represents a significant gap in women's health.^11^

### Medical school course outlines do not adequately include women's health

Our findings in keeping with a 2013 survey of American medical schools ascertained that 70% did not have a formal sex- and gender-integrated curriculum.^31^ This was supported by analysis of the curriculum at one such medical school, where it was determined that images and curricula did not represent the US population by sex/gender or ethnicity.^32^

Our findings suggest that Australian medical schools may lag behind other high-income countries in terms of incorporating women's health and gender equity into their course content. Yut-Lin et al. (2009) conducted a systematic review of the literature and demonstrated that medical schools in the United States and Canada have gender-sensitive medical school curricula, with set criteria and guidelines to address sex and gender stereotyping. This includes content, language, and processes that create gender sensitivity rather than perpetuate stereotypes.

Despite the findings of Yut-Lin et al.,^33^ assessment of medical students' sex and gender knowledge at one US university indicated significant gaps in their sex- and gender-based curricula and expressed a need for gender-based medicine to be further embedded and expanded into the curriculum.^34^

Our findings highlight the need for gendered content on medical school curricula, and there is growing evidence to support this. One university in the United States has developed an integrated longitudinal women's health curriculum emphasizing the social and biological differences between women and men and disease processes that are unique to women.^33^ Similarly, a medical school in Europe successfully integrated sex- and gender-based medicine into all course content from basic science to clinical assessments, including the differences in disease between females and males.^35^ Classes in gender medicine have been included in one Dutch medical schools' curricula since 2008 and this spurred a nationwide project to implement gender-based curriculum in all medical schools.^11^

The importance of gender medicine has also been acknowledged by governments in Sweden, Pakistan, the Philippines, Canada, and Australia^11^; however, in Australia, this acknowledgment does not appear to have filtered down into medical school curricula and course outlines.

### Clinical medicine textbooks do not account for sex and gender differences

Most textbooks described disease epidemiology as total numbers of females and males; however, in diseases with strong perceptions of a sex-based predominance, such as CHD, epidemiology was more likely to be sex segregated. One textbook provided total prevalence and female-only prevalence, which allows for inference of male prevalence, however, may also lead to a sex-specific diagnosis presumption and enhance sex- and gender-based preconceptions and ideologies. Otherwise, all other aspects of presentation, tests, treatment, and disease consequences were largely presented assuming females and males to be the same. This assumption is fundamentally flawed with medical textbooks, alongside other notable issues such as containing out-of-date information.^36^ Previous analysis of recommended medical textbooks also highlighted that a significant number of textbook conclusions are out of date or missing information from recently published studies.^36^

A review (1998) of select medical textbooks in Sweden determined that the male patient is presented as the norm and that gender stereotypes are perpetuated throughout the text and case examples.^21^ A sex/gender review of anatomy textbooks used by Australian medical schools determined that imagery remain predominantly male centered, and stereotyped roles such as the man in professional roles and the woman in domestic roles were reinforced.^30^

This study, however, did not find evidence of stereotyped images, perhaps reflecting progression of societal views on women, or perhaps a result of increased use of important test parameters such as ECGs and radiographs within clinical textbooks. Studies have long demonstrated the use of the male body in medical and anatomy textbooks, with potential consequences of medical students viewing the male body as the “norm” and additionally being less aware of female anatomy and less confident with examinations on women.^37,38^ Murciano-Guroff examined illustrations in general medical and general surgical textbooks, determining sex and gender subjectively based upon appearance of the faces, chests, and genitals and concluding that medical and surgical textbooks may continue to underrepresent females and advocating for more student exposure to female bodies.^39^

Morgan et al. examined 10 contemporary anatomy textbooks to determine if they are sex and gender neutral. Most anatomy textbooks used male illustrations and images as the standard for both surface and internal anatomy, and images of female anatomy were mostly limited to the breasts and reproductive organs. Some of the images depicting female anatomy were old-fashioned and lacking detail in a slightly older edition of the textbook. One textbook that contained clinical cases favored male examples as both doctor and patient; where female cases were provided, they were for conditions that are perceived to be associated with women such as varicose veins and osteoporosis, further perpetuating gender stereotypes in medicine, with impacts on how students and clinicians perceive women. This is evidenced in a survey of medical student perceptions and attitudes to sexism.

This survey demonstrated that a minority of students display overt sexism in the form of sexist remarks, or have witnessed overt sexism from senior clinicians, particularly related to anatomical sources.^11,40^ While many students claimed to be sensitive to sex and gender, they simultaneously failed to associate sexism with the negative aspects of sexism within anatomy teaching materials.^11^

Researchers examined the sex and gender content of eleven recommended medical textbooks in Dutch medical schools, investigating the content of cardiology/internal medicine, psychiatry, and pharmacology.^29^ In keeping with our findings, sex- and gender-specific information were lacking and there were few indications of sex and gender in the indices. Cardiology textbooks neglected specific mentions of women's health outside of single-line mentions. Psychiatry textbooks referred to the influence of hormonal fluctuations on depression, but otherwise sex- and gender-related information were also absent.

Safe levels of alcohol consumption for men and women were mentioned by one textbook and two textbooks briefly considered the higher vulnerability of women to alcohol. Pharmacology textbooks gave no sex- and gender-specific information, but did discuss interactions between oral contraceptives and other medications. Pharmacology textbooks, in keeping with clinical medicine textbooks, do not make it clear that the evidence base is derived from research that has historically excluded women.^29^ Analysis of gender representation in psychiatry textbooks found that women were more likely to represent diseases that have higher prevalence in females and males represent diseases with higher male prevalence. However, in diseases with equal or unknown prevalence, males were used in vignettes over females.^41^

### Importance of sex and gender representation in medical textbooks

Epidemiological studies have emphasized the differences in disease incidence and prevalence between women and men, and patient advocacy groups have campaigned for acknowledgment of sex and gender differences in disease.^42^ Failure to acknowledge and educate future clinicians on sex and gender differences in medicine and inadequate representation of women and women's health can perpetuate sexist stereotypes and maintain ideology of the male as the “norm” and the female as abnormal or “other.”

Neglecting sex- and gender-specific medicine in medical textbooks can have adverse effects on the knowledge base of medical students and subsequent adverse effects on women's health, including stereotyping and bias. There are numerous examples within the six diseases examined in this article of differences between women and men in the epidemiology, presentation, management, and outcomes of disease.^43^

Women have relatively higher morbidity, mortality, and poorer prognosis following ischemic cardiac events than men.^44^ Studies have demonstrated that women present with different, “atypical” symptoms of CHD, such as sharp pain, fatigue, shortness of breath, and indigestion.^1^ Descriptions in the textbooks of women's symptoms as “atypical” compared with men's is exemplary of the androcentricity in medicine and medical research.

Heart failure with preserved ejection fraction (HFwPEF) is almost doubly common in women than men and risk factors for this type of heart failure (hypertension and obesity) are more prevalent in women.^45^ HFwPEF is less responsive to standard heart failure medications than heart failure with reduced ejection fraction (HFwREF). Data demonstrate that women and men respond differently to angiotensin-converting enzyme inhibitors (ACE inhibitors), a frequently used heart failure medication, with women less likely to experience benefit from the drug unless they are appreciably symptomatic.^45^ In addition, women treated with digoxin for HFwREF had a higher death rate than women who took placebo; this was not observed in males in the trial,^45,46^ nor is it mentioned in any of the clinical textbooks analyzed in this study.

While IBS is twice as prevalent in women for those who seek health care, in the general population, the difference is less marked, suggesting women are more likely to seek help for their IBS than men, rather than necessarily being more likely to suffer IBS in the first instance.^47,48^ The effect of the menstrual cycle on IBS was absent from the clinical textbooks that were analyzed. Women report an increase in gastrointestinal symptoms around the time of menses compared with other stages of their cycle, commonly including loose stools, bloating, and pain.^47^ Female sex hormones are known to modulate gut motility and visceral pain.^49^ Studies have also demonstrated that IBS symptoms are inversely related to testosterone,^50^ meaning that hormonal and sex-based differences may be important in IBS pathogenesis and treatment.

Female sex is of significant influence on postoperative morbidity and mortality following peptic ulcer perforation, with one study demonstrating women patients requiring more postoperative ventilator support and experienced more renal failure than men.^51^ While symptoms, risk factors, and management appear to be the same for females and males, the textbooks do not state the increased risk of female sex, particularly for older women, for both peptic ulcer disease and the life-threatening complication of perforation.

CKD is more common in women than men in high-income countries, regardless of age,^52^ yet women are underrepresented in CKD clinics.^52^ Renal physiology differs between women and men. Female sex hormones affect the kidneys of women, increasing the synthesis of angiotensinogen, but decreasing renin and angiotensin-converting enzyme synthesis, impacting blood pressure regulation.^53^ Glomerular filtration rate (GFR) reference ranges may not be appropriate for women, yet all clinical textbooks assumed women and men require the same tests and treatment for CKD. Estimated GFR (eGFR) calculations are based on serum creatinine measurements, which are influenced by muscle mass. In the general adult population, women tend to have lower muscle mass, leading to lower creatinine measurements. This could lead to inaccurate eGFR recordings and thus inappropriate treatment for women.^53^

Women who are obese are at greater risk of developing renal calculi than women with a healthy body mass index, and the risk is higher for younger women.^54^ It is possible that different compositions of renal calculi are more prevalent by gender, for example, a recent study demonstrated that males are more likely to suffer uric acid stones, while females more likely to suffer calcium and magnesium calculi.^55^ These stones have different risk factors that were not apparent in the textbooks.

## Limitations

This study has a couple of limitations. First, the online content analysis constituted a desktop review of course outlines only and was restricted to information that was publicly available. We would recommend a further in-depth analysis of medical school curricula from across Australia to truly determine if women's health is omitted from medical school curricula outside of reproductive medicine. Second, we selected a sample of six common diseases as a representative sample to assess the presentation of women's health. This sample was guided by previous cross-sectional analyses and deliberately focused on medical specialties with low recruitment rates of women, compared with men, in clinical trials. This may have biased the results and it is possible that examination of other diseases would reveal more emphasis on women's health because of better inclusion in clinical trials.

Finally, it should be noted that throughout this article, the terms “female” and “women” are used at different points. This is owing to endeavors to keep terminology in keeping with the original source when referencing the work of others. The conflation of “female” and “women” throughout the literature can lead to confusion regarding the definition of women's health. For the purpose of this work, we consider women to be an inclusive term; however, there are notably large gaps in transgender health, which should also be addressed in future research.

## Conclusion

The important sex and gender differences in medicine are becoming increasingly known by researchers and clinicians, yet these are not reflected adequately in Australian medical school course outlines, curricula, or in clinical textbooks. This may have profound consequences on women's health in terms of diagnosis, time to diagnosis, rates of misdiagnosis, and response to tests and treatment, thus widening the sex and gender gap in clinical medicine.

No textbook discussed the significant socioeconomic differences in women and the impacts this can have on women's health. Clinical textbooks are a central recourse for medical students in Australia and omission of important sex and gender differences is a stark oversight. We recommend further in-depth analysis of medical school curricula in Australia and an urgent review of translation of research into clinical textbooks and guidelines to ensure sex and gender equity in clinical care. Enhancing awareness of sex and gender differences in medicine will enable physicians to treat males and females based upon their differing medical presentations and needs rather than assumptions and perceptions.

## Abbreviations Used

ACE inhibitors: angiotensin-converting enzyme inhibitors
CHD: coronary heart disease
CKD: chronic kidney disease
ECG: electrocardiogram
eGFR: Estimated GFR
GFR: Glomerular filtration rate
HFwPEF: Heart failure with preserved ejection fraction
IBS: irritable bowel syndrome
